# Clinical characteristics and antibodies against *Echinococcus granulosus* recombinant antigen P29 in patients with cystic echinococcosis in China

**DOI:** 10.1186/s12879-022-07597-8

**Published:** 2022-07-12

**Authors:** Jia Tao, Xiancai Du, Kejun Liu, Chan Wang, Yongxue Lv, Minglei Wang, Zhiqi Yang, Jihui Yang, Shasha Li, Changyou Wu, Minghao Li, Wei Zhao

**Affiliations:** 1 School of Basic Medical Sciences, Ningxia Medical University, Xingqing, Yinchuan, 750003 Ningxia Hui Autonomous Region China; 2 The Third School of Clinical Medicine, Ningxia Medical University, Jinfeng, Yinchuan, Ningxia Hui Autonomous Region 750021 China; 3 Ningxia Key Laboratory of Prevention and Control of Common Infectious Diseases, Xingqing, Yinchuan, 750003 Ningxia Hui Autonomous Region China; 4 Department of Hepatobiliary Surgery, General Hospital of Ningxia Medical University, Xingqing, Yinchuan, 750003 Ningxia Hui Autonomous Region China; 5 Department of Radiology, General Hospital of Ningxia Medical University, Xingqing, Yinchuan, 750003 Ningxia Hui Autonomous Region China; 6 Department of Hepatobiliary Surgery, People’s Hospital of Ningxia Hui Autonomous Region, Jinfeng, Yinchuan, 750021 Ningxia Hui Autonomous Region China; 7 Institute of Immunology, Zhongshan School of Medicine, Sun Yat-Sen University, Guangzhou, 5102275 Guangdong China

**Keywords:** *Echinococ*cus *granulosus*, *Hydatid disease*, Clinical characteristics, Recombinant antigen P29, Immunoglobulin

## Abstract

**Objectives:**

Cystic echinococcosis (CE) is a neglected parasitic zoonotic disease caused by the larval stage of the tapeworm *Echinococ*cus *granulosus* (*E. granulosus*). This study aimed to understand the clinical characteristics of human CE in Ningxia Hui Autonomous Region (NHAR) located in northwest China and to investigate the antibody profiles against the recombinant *E. granulosus* antigen P29 (r*Eg*.P29) in plasma of CE patients.

**Methods:**

A total of 37 human CE patients, along with 37 healthy donors enrolled in this study and demographic and clinical data were analyzed, including age, gender, laboratory data, symptoms, and cysts description. Plasma levels of cytokines, total IgG, and total IgE were determined by sandwich ELISA kits. Specific antibodies against r*Eg*.P29 and hydatid cyst fluid (HCF) were assessed by indirect ELISA.

**Results:**

The results revealed that females have a higher percentage of CE patients than males. The incidence of CE reached a peak in the 41–50 years-old group. The liver was the most frequent location, accounting for 91.9%. Based on the CT images, cysts of 34 patients who had liver involvement, were classified as 1 (2.9%) CE1, 12 (35.3%) CE2, 5 (14.7%) CE3a, 1 (2.9%) CE3b, and 15 (44.2%) CE5. Twenty-nine (78.4%) patients had a single cyst and 8 (21.6%) had at least two cysts. The most frequently reported symptom was upper abdominal pain. The plasma level of IL-6 and total IgE were significantly increased in CE patients compared with healthy donors. Additionally, IgG response to r*Eg*.P29 in CE patients was significantly higher than in healthy donors, and the dominant IgG subclass was IgG4. Further analysis of different patient groups revealed that r*E*g.P29-specific IgG and IgG4 were only elevated in CE patients with CE2 type cysts.

**Conclusions:**

This study systematically investigated the clinical characteristics of patients with CE and may provide a reference basis for the diagnosis and treatment of CE in NHAR. Furthermore, tests of specific IgG and IgG4 against r*Eg*.P29 can be used as an assisted method for imaging techniques to identify cystic activity and determine the best therapeutic approach for CE.

## Introduction

Cystic echinococcosis (CE), also known as hydatid disease, is a complex disease in humans caused by the larval stage of the tapeworm *E. granulosus*, and it is one of the 17 neglected tropical diseases recognized by the World Health Organization (WHO) [1]. The *E. granulosus* completes its life cycle in two hosts, a definitive carnivore host and an intermediate herbivore host. Human is an accidental intermediate host and becomes infected by ingesting food or water contaminated with the parasite’s eggs. After ingestion, the eggs hatch in the small intestine and become activated six-hooked oncospheres which penetrate through the mucosa and migrate through the circulatory system into internal organs where the oncospheres develop into hydatid cysts [2, 3]. The most commonly affected organ is the liver, followed by the lung, and all the other organs of the human body may be involved [4]. CE is endemic in many parts of the world, showing priority in agricultural and pastoral areas. The prevalence of CE ranges from 1 to 7% in community-based studies and 0 to 32 cases per 100,000 in hospital-based studies [5]. The estimated average number of global disability-adjusted life years (DALYs) was 285,407 (95% CI, 218,515–366,133) [6]. The mortality rate of CE is around 2%, but it may increase considerably in untreated or inadequately treated patients [7]. However, because the hydatid cyst grows slowly for several years and the initial phase of the primary infection is mostly asymptomatic, most patients miss the best treatment stage and get space-occupying lesions. These may lead to lethal complications, such as cyst rupture, possible anaphylactic shock, the spread of new cysts, bacterial infection, and even death [8].

Based on the ultrasonographic features, the hydatid cysts are differentiated into five types using the WHO-IWGE standard: CE1, CE2, CE3 (a, b), CE4, and CE5. The hydatid cyst type is used to assess their stage of development. Type CE1 and CE2 cysts are considered active cysts and usually fertile with viable protoscoleces; type CE3a and CE3b cysts are in a transitional stage; type CE4 and CE5 cysts are inactive and degenerative cysts [9]. The long-period survival of the hydatid cysts suggests they have defense mechanisms against the host immune response. The main feature of the relationship between the host and *E. granulosus* is the coexistence of both cellular and humoral responses. However, definite immune responses in CE patients are still complex and controversial. It is generally accepted that patients with CE have a mixed Th1/Th2 response. The Th1-dominated immunity in CE is related to protective immunity and well-response to chemotherapy. However, Th2 is associated with susceptibility to the disease and no response to chemotherapy [10, 11].

Several antigenic proteins in the hydatid cyst fluid have been reported to play key roles in immunoregulation mechanisms in patients with CE. *E. granulosus* P29 protein is absent in hydatid cyst fluid, but it is restricted to the metacestode stage of the parasite and the germinal layer of the cyst [12]. There are only a few previous studies conducted to investigate the immune response to *E. granulosus* P29 protein in humans. Two studies reported that P29 protein is a potential serological marker for the follow-up of human CE, especially in young patients [13, 14]. Our previous results showed that P29 protein induced high levels of specific IgG in vaccinated mice, and IgG1 and IgG3 were the dominant IgG subclasses. Furthermore, P29 protein showed a high protective immunity up to 96.6% against *E. granulosus* secondary infection in mice [15]. The P29 protein also showed high immunoprotection in sheep and induced Th1 and Th2 immune responses [16]. However, there are few studies that focus on the immune response induced by P29 protein in CE patients.

CE is highly endemic in the Ningxia Hui Autonomous Region (NHAR), located in northwest China [17]. In the current study, we collected and analyzed demographic and clinical information of human CE patients who were subjected to surgical operations in NHAR. Next, this study was used to investigate the specific immune responses to *E. granulosus* P29 protein in human CE patients. All assays were also performed using hydatid cyst fluid antigen (HCF), and the results were compared.

## Methods

### Study population

**Fig. 1:**
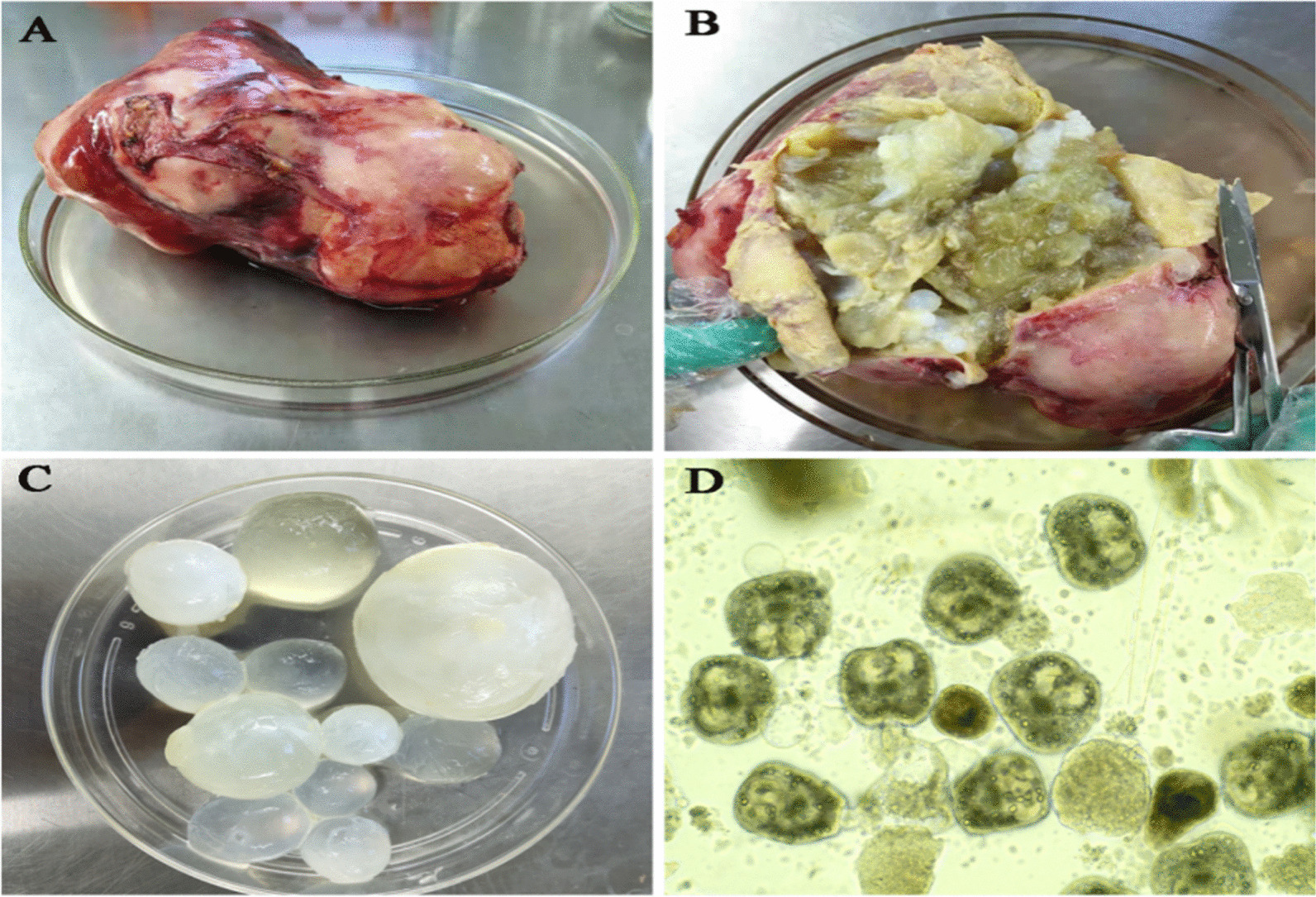
Images of the excised hydatid cyst in the liver of a 55-year-old woman (Patient P61) from Ningxia Hui Autonomous Region, northwest China. **A** Complete hydatid cyst after surgical resection. **B** Open hydatid cyst with daughter cysts and necrosis. **C** Daughter cysts. **D** Photomicrograph of protoscoleces isolated from the “daughter”cysts (original magnification, × 20)

CE patients participating in this study were enrolled in the two largest hospitals in the Ningxia Hui Autonomous Region (NHAR) in China from May 2020 to March 2022. The demographic and clinical data of the patients, including age, sex, length of hospitalization, medical history of hydatidosis, laboratory data, symptoms, and cyst description were collected. The CE patients involved in this study were subjected to surgical operations and confirmed by histopathology. A hydatid cyst was shown in Fig. 1. CE cysts were classified into five types based on computed tomography (CT): CE1, CE2, CE3 (CE3a and CE3b), CE4, and CE5. Twenty two and fifteen healthy donors were from epidemic regions in NHRA and our laboratory, respectively. All of healthy donors were healthy people with negative serology for CE.

### Plasma collection

Ten milliliters venous blood was drawn into heparin-anticoagulated tubes and plasma was obtained after centrifugation at 2500 rpm for 5 min at room temperature. Plasma samples were stored at – 80 ℃ until further analysis.

### Serological testing

The serological test was performed using a commercial enzyme-linked immunosorbent assay (ELISA) kit (Haitai, China) for *E. granulosus* IgG antibody detection according to the manufacturer’s instructions. Sample Index (SI) was calculated and interpreted for each plasma according to the manufacture’s instructions; ELISA was considered positive for SI > 1.1, negative for SI < 0.9, and equivocal for 0.9 < SI < 1.1.

### Cytokines, total IgG, and total IgE determination

Cytokines including IFN-γ, IL-2, IL-4, TNF-α, IL-10, and IL-6 were determined by sandwich ELISA kits (BD Biosciences, USA) according to the manufacturer’s instructions. The optical density (OD) values were transformed into concentrations (pg/ml) according to the cytokine-specific standard curves. The lower limits of cytokine detection are 4.7 pg/ml for IFN-γ and IL-6, and 7.8 pg/ml for TNF-α, IL-2, IL-4, and IL-10. Total IgG from plasma samples was assessed using a commercial enzyme-linked immunosorbent assay (ELISA) kit (Meimian, Jiangsu, China). Total IgE in human plasma was determined using the IgE Human Uncoated ELISA kit (Invitrogen, Austria), and calculated as absolute concentrations according to the standard curve.

### Preparation of r*Eg*.P29 and hydatid cyst fluid (HCF) crude antigens

Recombinant plasmid P29/pET28a was constructed and converted into *E. coli* BL21 pLysS in our laboratory before [15]. Hydatid cyst fluid (HCF) crude antigen was collected in cysts of patients with hepatic cyst echinococcosis. Fluid was aspirated from the cyst using a 5 ml syringe and centrifuged at 1000*g* at 4 ℃ for 30 min. The supernatant was used as HCF crude antigen. Protein concentration was measured with a BCA Protein Assay Kit (KeyGEN, China) and stored at − 80℃.

### r*Eg*.P29 and HCF specific antibodies measurements

Specific anti-r*Eg*.P29 IgG, IgM, IgA, and IgE in plasma samples of patients with CE and healthy donors were assessed by indirect ELISA. Briefly, ninety-six-well polystyrene plates (Corning, USA) were coated with 100 μl/well of r*Eg*.P29 antigen at 10 μg/ml and HCF at 0.6 μg/ml in coating buffer (0.1 M carbonate buffer, pH 9.6) overnight at 4 ℃. Following five washes with PBS containing 0.05% (v/v) Tween-20 (PBS-T), 200 μl of the blocking buffer (5% skimmed milk in PBST, w/v) were added in each well, and plates were incubated at 37 ℃ for 2 h. After washing the plates five times with PBST, 100 μl of plasma samples were added at 1:500 dilution (1:100 for IgE testing) in blocking buffer and incubated at 37 ℃ for 1 h. Following five times washes, plates were incubated with 100 μl of peroxidase-conjugated goat anti-human IgG (dilution 1:5000 in blocking buffer), goat anti-human IgM (dilution 1:8000 in blocking buffer), goat anti-human IgA (dilution 1:2000 in blocking buffer), goat anti-human IgE (dilution 1:1000 in blocking buffer) and incubated at 37 ℃for 1 h. Subsequently, 100 μl of TMB substrate buffer (Solabo, China) was added to each well and incubated at 37 ℃ for 10 min in the dark. Finally, the reaction was terminated with 50 μl stop buffer (Solabo, China), and the absorbance of each well was read at 450 nm and referenced to 630 nm by a microplate reader. All samples were tested in triplicate. HRP conjugated antibodies against IgG, IgM, IgA, and IgE were acquired in abcam, USA.

To determine IgG subclasses, r*Eg*.P29 and HCF coated plates were incubated with 100 μl of 1:100 diluted samples in blocking buffer. Horseradish peroxidase (HRP) labeled anti-human IgG1, IgG2, IgG3, and IgG4 conjugates (all in dilution 1:100) in blocking buffer were added to each well. HRP conjugated antibodies against IgG subclasses were acquired in Invitrogen, USA.

### Statistical analysis

Category variables were expressed as absolute frequencies and percentages, whereas continuous parameters were expressed as mean and standard deviations. A Mann–Whitney U test was used to compare the differences between the two groups. A Kruskal–Wallis H rank-sum test was applied to compare continuous variables for multiple groups. Statistical analyses were performed using GraphPad Prism software version 8 (GraphPad Software Inc., San Jose, CA, USA); a two-tailed *p*-value < 0.05 was considered to be statistically significant.

## Results

### Demographical and clinical characteristics of the population

Demographical information of CE patients and healthy donors are shown in Table 1. Thirty-seven patients with CE were enrolled in this study and their mean age was 50.9 years. The incidence of CE reached a peak in the 41–50 years-old group. Among the patients in this study, 22 (59.5%) were newly diagnosed and 15 (40.5%) suffered a relapse. For relapsed patients, the mean years of duration for CE were 17.7 years, ranging from 8 to 50 years.

**Table 1:** Demographical information of 37 CE patients and 37 healthy donors (HD) from Ningxia Hui Autonomous Region, northwest China

	CE (n/%)	HD (n/%)
Age, years (mean ± SD)	50.9 ± 13.2	44.2 ± 14
< 30	1 (2.7)	8 (21.6)
31–40	7 (18.9)	9 (24.3)
41–50	12 (32.4)	7 (18.9)
51–60	8 (21.6)	7 (18.9)
61–70	4 (10.9)	6 (16.3)
> 70	5 (13.5)	0 (0)
Gender		
Female	24 (64.9)	21 (56.8)
Male	13 (35.1)	16 (43.2)
Hospital stay, days (mean ± SD)	14.4 ± 4.2	–

**Fig. 2:**
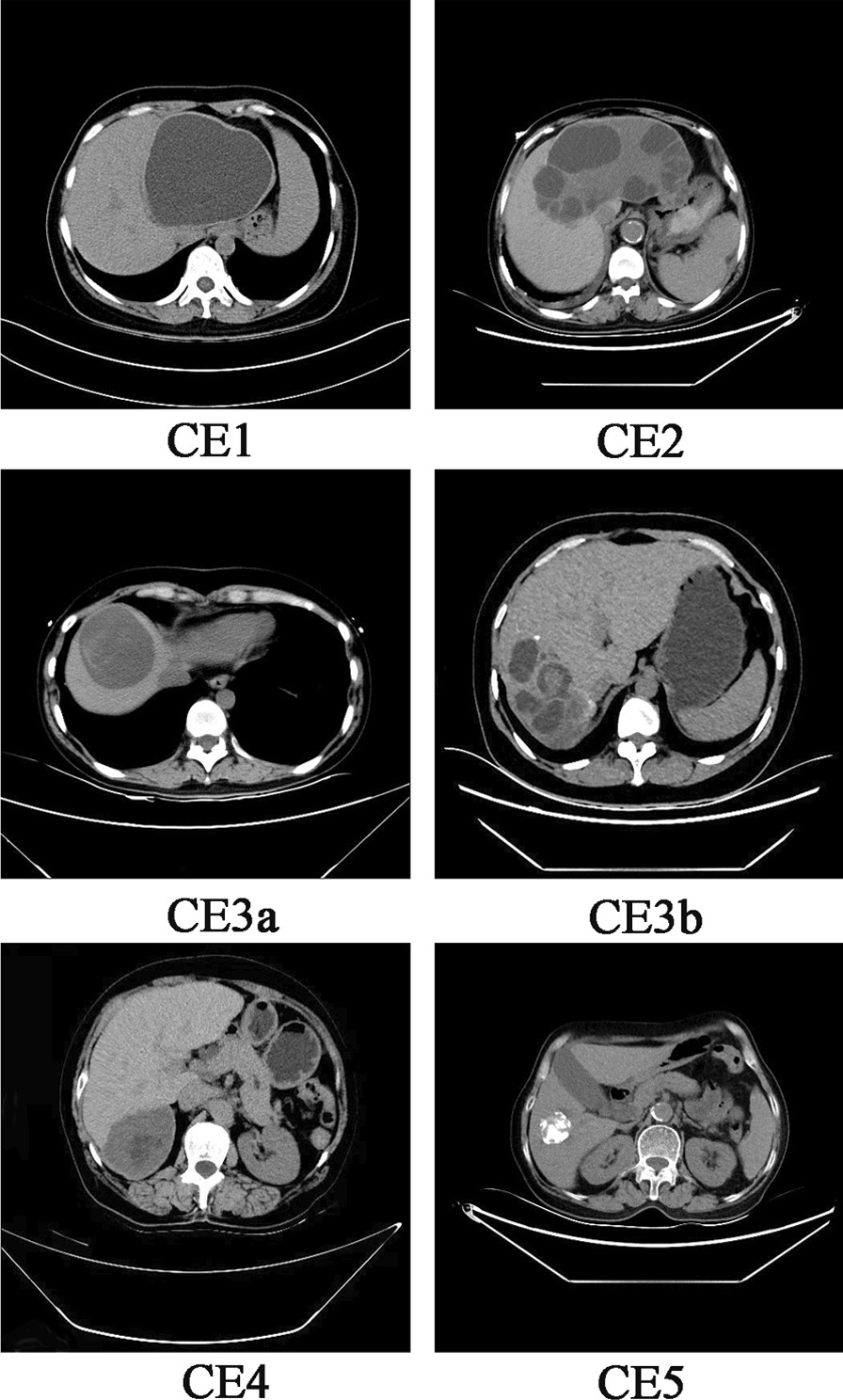
CT images of different type of hydatid cysts in patients from Ningxia Hui Autonomous Region, northwest China. CE1: unilocular cyst with liquid content, CE2: multivesicular, multiseptated cysts, the cysts are well delineated, smooth with uniform content, CE3a: cysts with liquid content and detached endocyst, CE3b: cysts with daughter cysts inside a mucinous or solid cyst matrix, CE4: heterogenous solid cysts with degenerative content and CE5: cysts with degenerative content and heavily calcified wall

In 30 (81.1%) CE patients, the disease was limited to the liver, and the right lobe was the most frequent location. Based on the computed tomography (CT) results of 34 patients with liver echinococcosis, the cysts were classified into different types, and Fig. 2 shows the CT images of different types. Based on the WHO classification, among 46 cysts from 35 patients, 8 (17.4%) cysts were small, with a diameter of less than 5 cm; 24 (52.2%) were medium-sized, with a diameter ranging from 5 to 10 cm; and 14 (30.4%) cysts were large (> 10 cm). The characteristics of hydatid cysts are shown in Table 2.

**Table 2:** Characteristics of hydatid cysts of 37 CE patients from Ningxia Hui Autonomous Region, northwest China

Characteristics	N / %
Cyst location (n = 37)	
Liver	
Right lobe	24 (64.9)
Left lobe	4 (10.8)
Both lobes	2 (5.4)
Liver and Lung	1 (2.7)
Liver and spleen	1 (2.7)
Liver and abdominal cavity	2 (5.4)
Lung and ribs	1 (2.7)
Spleen	1 (2.7)
Pelvic abdominal cavity	1 (2.7)
Number of lesion (n = 37)	
Single	29 (78.4)
Multiple	8 (21.6)
Cyst dimension (n = 46)	
Large	14 (30.4)
Medium	24 (52.2)
Small	8 (17.4)
Classification of cyst types (n = 34)	
CE1	1 (2.9)
CE2	12 (35.3)
CE3a	5 (14.7)
CE3b	1 (2.9)
CE5	15 (44.2)

Among all 37 patients, 8 (21.6%) were asymptomatic; among them, 3 and 5 patients found cysts during postoperative annual ultrasound examination and physical examination, respectively. For symptomatic patients, the most frequently reported symptom was upper abdominal pain, which was observed in 24 (64.9%) patients. Other symptoms were as follows: nausea, vomiting in 8 patients, lower back pain in 2, loss of weight in 3, and respiratory symptoms in 1.

The serology test was scored positive in 27 patients, equivocal in 3 patients, and negative in 7 patients, respectively. All healthy donors are negative for the serological test. Laboratory hematological analysis showed 5 patients had an eosinophil increase, 13 patients had a monocyte increase, 3 patients had erythropenia, and 5 cases had hemoglobinia. Decreased levels of TP, ALB, and A/G ratio were observed in 13, 17, and 11 patients, respectively. An increase in alanine aminotransferase (ALT) level in 7 patients, aspartate transaminase (AST) in 8 patients, γ-glutamyl transferase (GGT) in 7 patients. Notably, AST/ALT ratio was increased in 16 patients.

### Cytokines, total IgG, and IgE in plasma samples of two groups

**Fig. 3:**
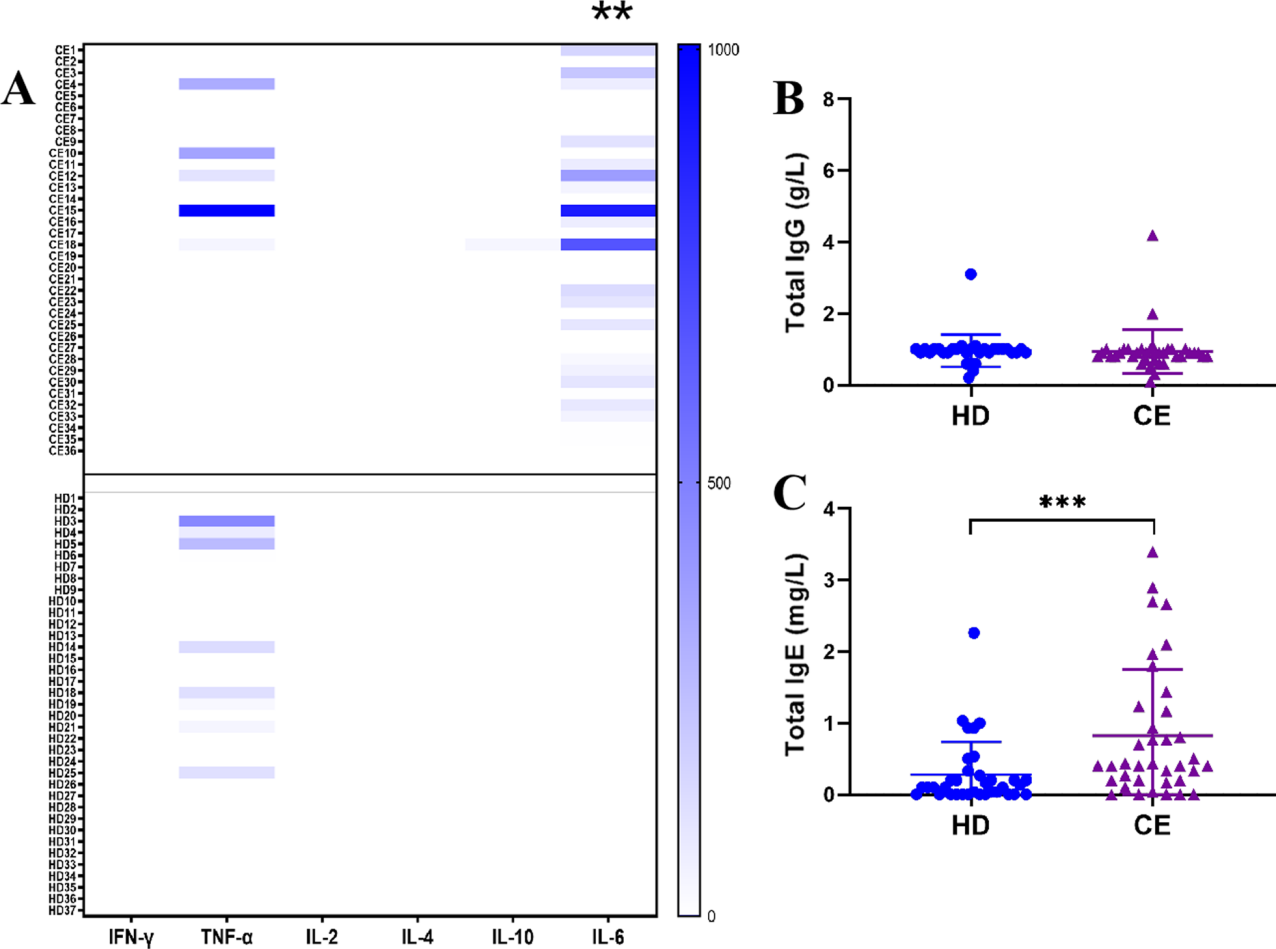
Quantification of cytokines, total IgG, and total IgE in plasma samples. **A** A heatmap showing 6 cytokines in plasma. Healthy donors (HD), n = 37; CE, n = 36. Levels of total IgG (**B**) and total IgE (**C**) in plasma samples. **: *p* < 0.01,***: *p* < 0.001. HD, n = 37; CE, n = 37

Plasma levels of IFN-γ, TNF-α, IL-2, IL-4, IL-10, and IL-6 were measured, Fig. 3A. The plasma level of IL-6 was significantly increased in CE patients compared with healthy donors (*p* < 0.01). TNF-α exceeded the first standard of the ELISA in only 5 patients and 8 healthy donors. However, there was no significant difference in plasma levels of TNF-α between the CE (51.12 ± 182.6 pg/ml) and HD (34.85 ± 93.26 pg/ml) groups. Levels of IFN-γ, IL-2, and IL-4 in all enrolled subjects were very low and below detection limits. Similarly, the concentration of IL-10 was only detected in one patient. No significant difference in total IgG levels was found between the CE (0.94 ± 0.62 g/L) and HD (0.96 ± 0.45 g/L) groups (Fig. 3B). The total IgE levels were significantly higher in CE group (0.83 ± 0.92 mg/L) than in the HD group (0.28 ± 0.46 mg/L) ( *p* < 0.001, Fig. 3C).

### Antibodies against r*Eg*.P29 and HCF in patients with CE

**Fig. 4:**
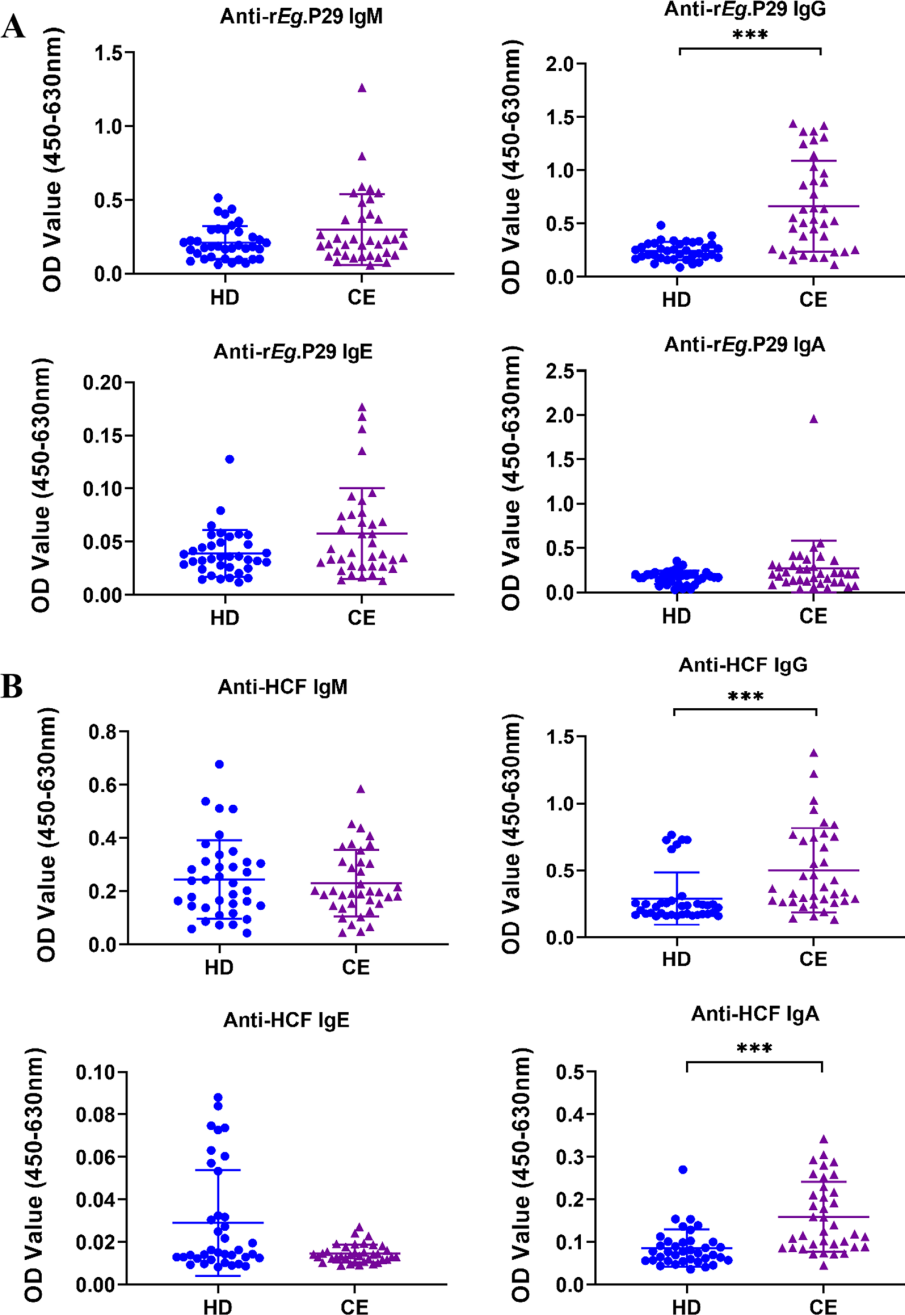
Specific antibodies against r*Eg*.P29 and HCF in plasma samples of CE patients and healthy donors (HD) from Ningxia Hui Autonomous Region, northwest China. The optical density (OD) values of the anti‐r*Eg*.P29 (**A**) and anti‐HCF (**B**) IgM, IgG, IgE, IgA. Two groups were compared by the Mann–Whitney test (***: *p* < 0.001). HD, n = 37; CE, n = 37

To understand whether *E. granulosus* infection induces humoral immune responses to r*Eg*.P29 and HCF, we assessed anti-r*Eg*.P29 and anti-HCF IgM, IgG, IgA, and IgE in plasma from patients with CE and healthy donors by indirect ELISA. The results showed that specific IgG against r*Eg*.P29 was higher in CE patients compared to HD individuals (*p* < 0.001), while no statistically significant differences were observed in IgM, IgA, and IgE (Fig. 4A). For HCF, specific IgG and IgA antibodies were significantly higher in the plasma of the CE group than in the HD group (*p* < 0.001), but there was no significant difference in IgM and IgE (Fig. 4B).

**Fig. 5:**
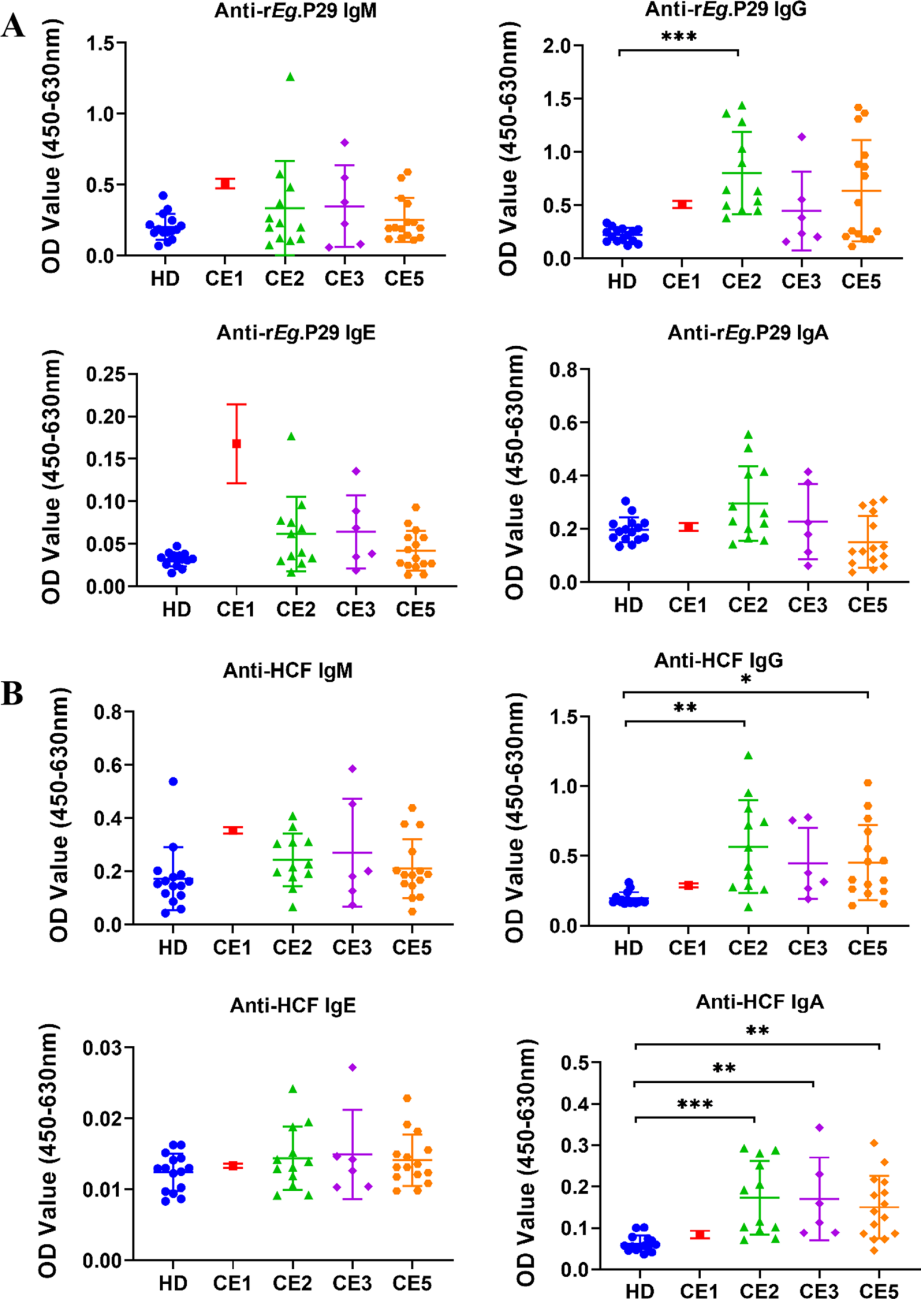
Comparison of specific antibodies against r*Eg*.P29 and HCF from plasma of patients with different types of cysts and healthy donors (HD) from Ningxia Hui Autonomous Region, northwest China. The optical density (OD) values of the anti‐r*Eg*.P29 (**A**) and anti‐HCF (**B**) IgM, IgG, IgE, and IgA. Multiple groups were compared by the non-parametric Kruskal Wallis test (*: *p* < 0.05; **: *p* < 0.01; *** *p* < 0.001). HD, n = 15; CE1, n = 1; CE2, n = 12; CE3, n = 6; CE5, n = 15

According to the type of cysts, the CE patients were divided into different groups to compare the antibody profiles. Specific IgG response to r*Eg*.P29 only in the CE2 group was higher than in the HD group (*p* < 0.001) (Fig. 5A). Anti-HCF IgG was significantly higher in both CE2 (*p* < 0.01) and CE5 (*p* < 0.05) groups than in the HD group. The levels of IgA against HCF were significantly higher in all CE2 (*p* < 0.001), CE3 (*p* < 0.01), and CE5 (*p* < 0.01) groups than in the HD group (Fig. 5B).

### IgG subclasses to r*Eg*.P29 and HCF in patients with CE

**Fig. 6:**
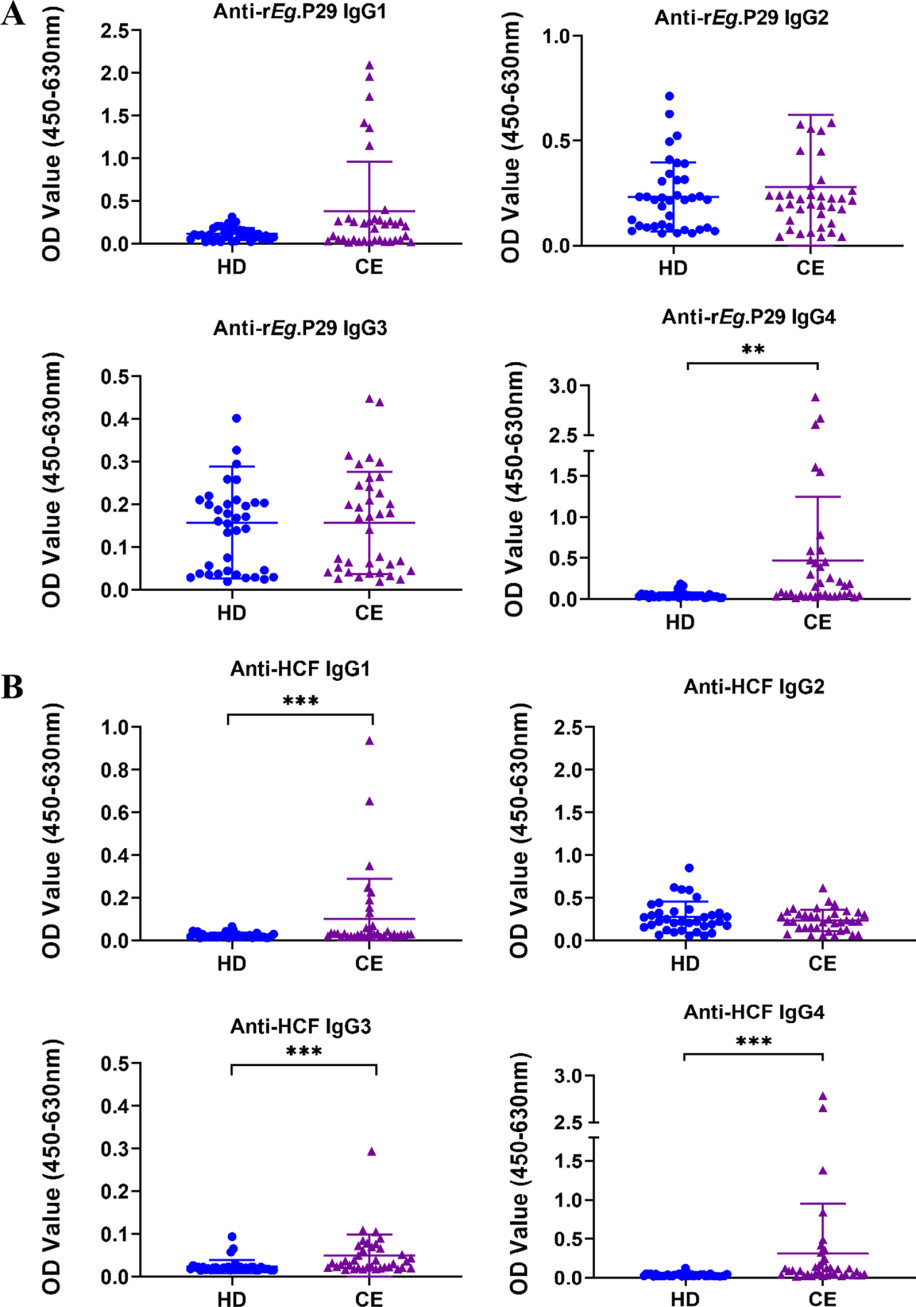
IgG subclasses against r*Eg*.P29 and HCF from plasma of CE patients and healthy donors (HD) from Ningxia Hui Autonomous Region, northwest China. The optical density (OD) values of the anti‐r*Eg*.P29 (**A**) and anti‐HCF (**B**) IgG1, IgG2, IgG3, IgG4. Two groups were compared by the Mann–Whitney test (**: *p* < 0.01,***: *p* < 0.001). HD, n = 22; CE, n = 27

**Fig. 7:**
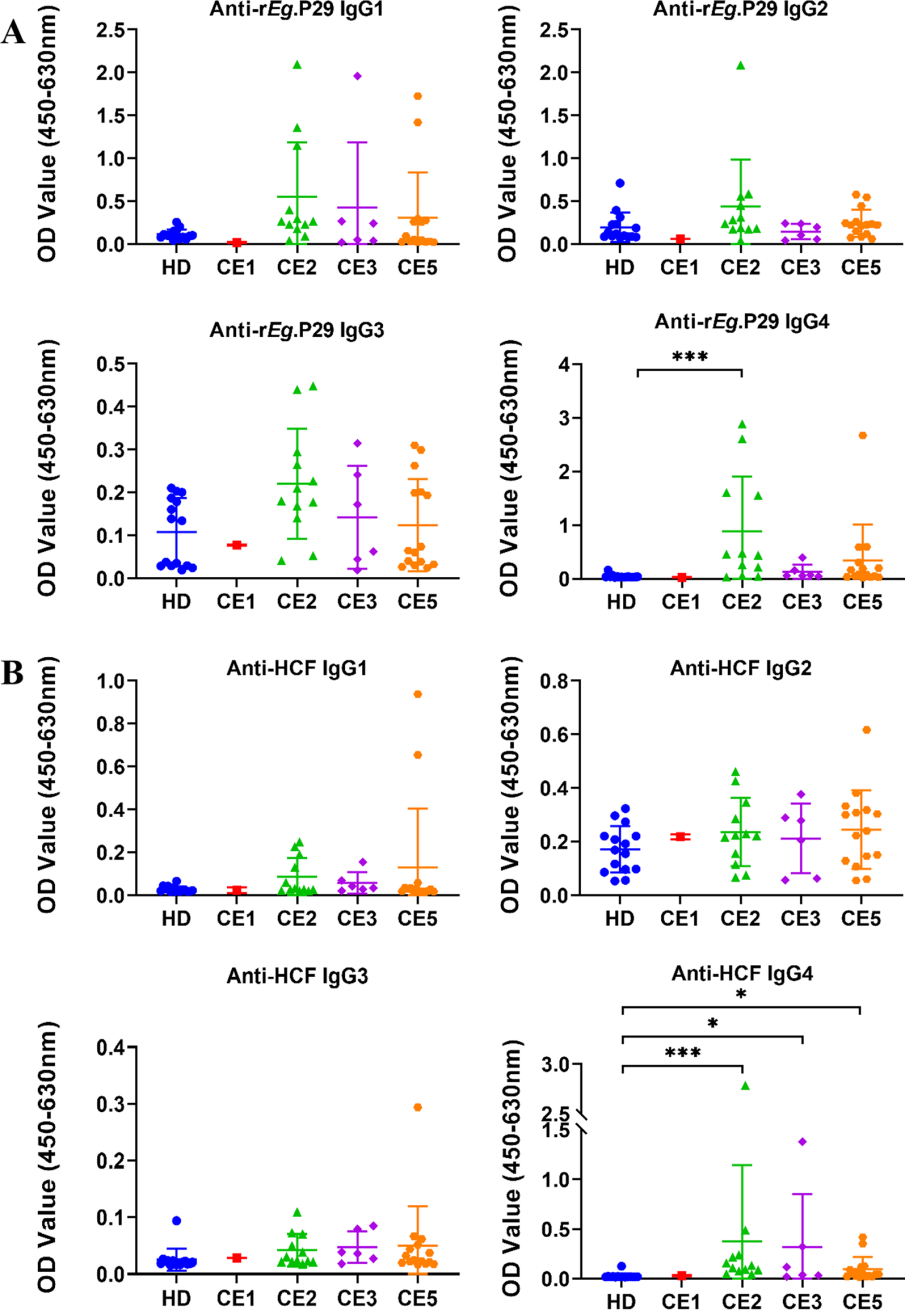
Comparison of IgG subclasses against r*Eg*.P29 and HCF from plasma of patients with different types of cysts and healthy donors (HD) from Ningxia Hui Autonomous Region, northwest China. The optical density (OD) values of the anti‐r*Eg*.P29 (**A**) and anti‐HCF (**B**) IgG1, IgG2, IgG3, IgG4. Multiple groups were compared by the non-parametric Kruskal Wallis test (*: *p* < 0.05; **: *p* < 0.01; ***: *p* < 0.001). HD, n = 15; CE1, n = 1; CE2, n = 12; CE3:, n = 6; CE5, n = 15

To further characterize the IgG response against r*Eg*.P29 and HCF, IgG subclasses were tested in CE patients and healthy donors. The specific IgG4 subclass against r*Eg*.P29 was significantly higher in the plasma of CE patients in comparison to HDs (*p* < 0.001), while no differences were observed between the two groups in IgG1, IgG2, and IgG3 (Fig. 6A). Of interest, IgG subclasses to HCF had a different pattern from the profiles of r*Eg*.P29 (Fig. 6B). CE group showed significantly higher IgG1, IgG3, and IgG4 reactivity to HCF compared with the HD group (*p* < 0.001). HCF-specific IgG2 subclass was barely detectable in the two groups. Furthermore, we compare the specific IgG subclasses to r*Eg*.P29 and HCF in different patient groups. The results showed that IgG4 against r*Eg*.P29 was only significantly higher in the CE2 group than in the HD group (*p* < 0.001), but there was no significant difference between other patient groups and the HD group (Fig. 7A). Meanwhile, CE2 (*p* < 0.001), CE3 (*p* < 0.05), and CE5 (*p* < 0.05) groups showed significantly higher IgG4 reactivity to HCF compared with the HD group (Fig. 7B).

## Discussion

Human CE is a complex parasitic zoonotic disease endemic to rural areas, especially in the Mediterranean Basin, northern and eastern Africa, South America, Eastern Europe, Australia, Russia, and China [18–20]. CE is mainly prevalent in the northwest part of China, including Gansu Province [21], Xinjiang Uigur Autonomous Region [22], Qinghai Province [23], Tibet Autonomous Region [24], Sichuan Province [19], Heilongjiang Province [25] and Ningxia Hui Autonomous Region [26]. CE is a serious public health problem for the NHAR. It is reported that different CE patient groups showed significant differences in their demographic and clinical characteristics [27].

In our study, females with CE had a higher occurrence than males. Many previous studies reported similar results [25, 27, 28]. Meanwhile, other studies showed the prevalence of males was higher than that of females [29–31]. This result in our study could be explained by the fact that females are not only involved in farming work but also hold more home activities, such as preparing contaminated raw vegetables or meat or feeding the animals in the household. The mean age of CE patients was 50.9 years, and the incidence was highest in the age group of 41–50 years (32.4%). Similar observations were reported in Heilongjiang province in China (27.32%) [25], Tunisia (22.72%) [32] and Turkey (22.62%) [33]. The reason for this may be that patients with CE are always asymptomatic for years, despite the fact that the causal infection is acquired in early life. Previous studies have reported that hydatid cysts can localize in any organ of the human body and the liver is the most affected organ, followed by the lungs [34, 35]. Similarly, our results showed that the liver is affected in 91.9% of the cases, and the right lobe is more frequently affected than the left. In 2 of the 37 patients with pulmonary CE, both of them with another affected organ, one for the liver and one for the ribs. We also found hydatid cysts located in the spleen and pelvic abdominal cavity. Contrary to most of the studies, a small number of studies reported that the most affected organ was the lung [20, 36]. This may be due to differences in geographical factors, the genotype of *E. granulosus*, genetic predisposition, or co-infections with other pathogens, such as HIV and/or TB. In the present study, most of the patients harbored a single cyst (70%), the remaining patients with at least 2 cysts. Similarly, in another study in China, 75.96% and 24.04% of hepatic CE patients had a single cyst and multiple cysts [25]; in Mongolia, 78.1% of hepatic CE patients had a single cyst, 20% had 1–3 cysts, and 1.3% had more than 3 cysts [37]; in Tunisia, a single cyst was in 83.57% and multiple cysts in 16.43% of CE patients, respectively [32]. This indicates that most primary infections in humans consist of a single cyst. The diameter of 46 cysts from 35 patients ranged from 2 to 20 cm, and 17.4%, 52.2%, and 30.4% of cysts were small, medium, and large, respectively. The results showed that most patients had large or medium-sized cysts in this study. The small cysts do not induce major pathology, and patients may have an asymptomatic disease course for years. So, most echinococcal cysts are diagnosed incidentally during physical examination or for other reasons. However, in this study, most patients didn’t have regular physical examinations due to living in rural areas. They are diagnosed when the cysts grow large enough to cause symptoms. Previous studies demonstrated that cysts’ size can influence the treatment duration and effectiveness of anti-hydatid treatment [38, 39]. Patients with small cysts were more efficacious for anti-hydatid treatment and needed the shortest curative treatment period. Therefore, it is important to perform regular surveillance of echinococcosis in endemic areas, even in remote communities.

In the analysis of symptoms of CE patients in the present study, upper abdominal pain was the most common symptom in 64.9% of the patients, which is consistent with findings of previous studies [5]. Other symptoms include vomiting, lower back pain, and loss of weight. One patient with a pulmonary hydatid cyst had respiratory symptoms. Similar to other studies, 8 (25.9%) of our patients were asymptomatic at diagnosis, and among them, 2 relapsed cases were identified in the postoperative annual ultrasound examination, and the remaining 6 newly-diagnosed patients were observed during physical examination. Previous studies revealed the recurrence rate after surgery varied between 1.5 and 6.3% [40]. According to Gulsun et al. [41], recurrence cases were observed 3 years following surgery. Another study also reported that relapsed patients may have symptoms 3 to 4 years after surgery [42]. In our study, among the 15 relapsed cases, 3 and 2 patients had a second and third incidence of hydatidosis, respectively, and the time to recurrence ranged from 5 to 30 years. The recurrence was diagnosed after a lengthy time, perhaps because patients didn’t perform annual postoperative ultrasound exams. Therefore, these findings re-emphasize the need for long-term follow-ups over years for CE patients.

Since *E. granulosus* infections may lead to liver damage and inflammation, we further assessed the alteration of the routine blood tests, liver function tests, cytokines, and total antibodies in the plasma sample of CE patients. In the current study, elevation in eosinophil was present in 13.5% of cases, which was also consistent with a study conducted by Ebrahimipour et al. [43]. We also found that levels of total IgE and IL-6 were higher in CE patients than in healthy donors. There were positive correlations of eosinophil percentage with total IgE, IL-6, and TNF-α. TP, ALB, A/G ratio were decreased in 13 (35.1%),17 (45.9%), and 11 (29.7%) patients, respectively. The increase of ALT, AST, AST/ALT ratio, and GGT were found in 7(18.9%), 8 (21.6%), 16 (43.2%) and 7 (18.9%) cases. Notably, the percentage of patients with increased AST/ALT ratio was higher than ALT and AST. However, the relationship between CE and AST/ALT ratio remains further research.

Due to the CE cysts and host cohabiting for a long time, the immune response between the parasite and the host is complex and contradictory. Previous studies revealed that most patients develop a significant antibody response to *E. granulosus* cysts, with different antibody isotypes (IgG, IgM, IgA, and IgE). However, immune regulation mechanisms and the strategies of immune evasion in humans are still not clear [44]. *E. granulosus* P29 protein is a 29 kDa antigen that is absent in hydatid cyst fluid but localized to the protoscoleces and the germinal layer of the cyst [12]. To date, the research on immune responses against *E. granulosus* P29 protein in humans is quite limited.

In the present study, the level of IgG against r*Eg*.P29 was significantly higher in CE patients than in healthy donors, and the dominant IgG subclass is IgG4. Consistent with our findings, other studies reported that the IgG4 antibodies may play a prominent role in the responses to *E. granulosus* infection. Antigens-specific IgG4 may serve as a link between AgB and Th2 cell activation and could be used for serological diagnosis of CE [45, 46]. It has been reported that Th2 cytokines regulate the synthesis of IgG4. Our findings may indicate that r*Eg*.P29 elicits a Th2 cell-mediated immune response to induce IgG4 production. To date, an accepted view is that antibody responses in CE patients are correlated with the clinical stage and cysts activity. When the patients were divided into different groups based on the cyst types, we found levels of IgG and IgG4 against r*Eg*.P29 were significantly higher only in patients with CE2 type cysts compared with healthy donors. The results in our study were comparable to the previous understanding that IgG4 and IgG1 were mainly increased in patients with active and transitional cysts [47, 48]. There was only one case with CE1 type cyst, which was not statistically significant due to the small number of cases, and r*Eg*.P29-specific antibodies in the plasma of patients with CE3 and CE5 types were not statistically significant compared with healthy controls. This may be due to the high activity of the CE2 type cyst, which can secrete a large amount of antigenic material and induce a strong immune response, while the immunity of the body is weakened by the gradual decrease in the activity of the cysts from CE3 to CE5. Meanwhile, we assessed the antibody responses to HCF in CE patients. We found IgG and IgA against HCF were higher in CE patients, and the dominant IgG subclasses were IgG1, IgG3, and IgG4. Unlike our results, Aceti et al*.* reported a predominance of IgG1, IgG2, and IgG3 antibodies among sera from hydatid patients [49]. These differences in antibody profiles may be due to HCF containing different antigens that may lead to different responses.

## Conclusion

To our best understanding, this is one of few systematic study to investigate the clinical characteristics of patients with CE in NHAR. These results may provide a reference basis for the diagnosis and treatment of CE in the region. Our further study found that specific IgG and IgG4 against r*E*g.P29 was increased in the CE2 group compared with the HD group, but there were no significant differences of the CE3 and CE5 groups with the HD group. These findings suggest that tests based on IgG and IgG4 against r*Eg*.P29 together with imaging techniques, can be used for the identification of cystic activity, leading to the best therapeutic approach for CE. This study was conducted using a limited number of patients with unequal numbers of patients in different stages. Larger and further research is required to confirm our results.

## Data Availability

The datasets analysed during the current study are available from the corresponding author on reasonable request.
